# Patient satisfaction and shared decision-making in a psychiatric ward

**DOI:** 10.1192/j.eurpsy.2025.1600

**Published:** 2025-08-26

**Authors:** P. Tilelis, G. Papaefstathiou, K. Anargyros, E. Mpaka, I. Chatzidakis, T. Jupe, D. Kontis

**Affiliations:** 1 4th Inpatient Department, Psychiatric Hospital of Attica, Athens, Greece

## Abstract

**Introduction:**

In the dynamic setting of psychiatric care, patient satisfaction and shared decision-making are crucial for optimal, personalized treatment. This approach is particularly vital in the context of a psychiatric ward, where engaging patients in treatment decisions could enhance their satisfaction and the overall therapeutic outcomes.

**Objectives:**

This study evaluated the impact of shared decision-making on patient satisfaction from services among patients treated in a psychiatric ward. Additionally, it examined whether there are differences in satisfaction between psychotic and non-psychotic patients.

**Methods:**

53 patients (30 women and 23 men) who were hospitalized in the 4th Inpatient Department of the Psychiatric Hospital of Attica were assessed using the Client Satisfaction Questionnaire (CSQ-8) and the 9-Item Shared Decision-Making Questionnaire (SDM-Q-9). Diagnoses were based on the Mini International Neuropsychiatric Interview (M.I.N.I.). A multiple linear regression analysis was conducted to evaluate the effect of the SDM-Q-9 total score and of the presence of psychotic symptoms on the CSQ-8 total score. The relationship of separate SDM-Q-9 and CSQ-8 items, controlling for the presence of psychosis was further analyzed through partial correlation analyses. The Bonferroni method was employed in order to adjust for multiple comparisons.

**Results:**

The mean age of the participants was 47.49 years (SD=13,65), and the mean duration of the disease was 15 years (SD=12,36). Thirty-two patients were diagnosed with psychotic disorders (psychotic disorder=28, bipolar disorder=1, mood disorder with psychotic features=3), while twenty-one were diagnosed with non-psychotic disorders (including Major Depressive Disorder, Obsessive Compulsive Disorder, Bipolar Disorder and Substance Use disorders). Increasing SDM-Q-9 total score significantly correlated with increasing CSQ-8 total score (B=0,276, 95% CI=,177, 0.374, t=5,627, p<0.001). Partial correlation analyses showed that separate SDM-Q-9 items significantly correlated with separate CSQ-8 items, while many correlations survived the stringent bonferonni correction. The presence of psychotic symptoms was not associated with client satisfaction.

**Conclusions:**

Our findings suggest that shared decision-making correlates positively with patient’s satisfaction from services during their hospitalization in a psychiatric ward and this was independent of the presence of psychosis. In the ongoing effort to find real-world solutions in mental health, integrating shared decision-making practices into psychiatric care can be a fundamental strategy to empower and satisfy patients suffering from mental disorders.

**Disclosure of Interest:**

None Declared

